# Muscle Function Differences between Patients with Bulbar and Spinal Onset Amyotrophic Lateral Sclerosis. Does It Depend on Peripheral Glucose?

**DOI:** 10.3390/jcm10081582

**Published:** 2021-04-09

**Authors:** Jose Enrique de la Rubia Ortí, Jose Luis Platero Armero, Claudia Emmanuela Sanchis-Sanchis, Sandra Sancho-Castillo, Alejandro Salazar, Jordi Caplliure-Llopis, Esther Navarro-Illana, Carlos Barrios, Jesús Escribá-Alepuz, María Benlloch

**Affiliations:** 1 Department of Basic Medical Sciences, Catholic University of Valencia, 46001 Valencia, Spain; joseenrique.delarubi@ucv.es (J.E.d.l.R.O.); joseluisplateroarmero@gmail.com (J.L.P.A.); sandra.sancho@ucv.es (S.S.-C.); maria.benlloch@ucv.es (M.B.); 2 Department of Statistics and Operational Research, University of Cádiz, Puerto Real, 11510 Cádiz, Spain; alejandro.salazar@uca.es; 3 The Observatory of Pain, University of Cádiz, 11009 Cádiz, Spain; 4 Biomedical Research and Innovation Institute of Cádiz (INiBICA), 11009 Cádiz, Spain; 5 Doctoral Degree’s School, Catholic University of Valencia, 46001 Valencia, Spain; jorcallo@mail.ucv.es; 6 Institute for Research on Musculoskeletal Disorders, Valencia Catholic University, 46001 Valencia, Spain; carlos.barrios@ucv.es; 7 Neurophysiology Department, Sagunto University Hospital, 46520 Valencia, Spain; jesusescriba@hotmail.com; 8 Institute of Sleep Medicine, 46021 Valencia, Spain

**Keywords:** amyotrophic lateral sclerosis, glucose, alkaline phosphatase, spinal onset ALS, bulbar onset ALS

## Abstract

Background: One of the pathogenic mechanisms of ALS disease is perturbed energy metabolism particularly glucose metabolism. Given the substantial difference in the severity and the prognosis of the disease, depending on whether it has a bulbar or spinal onset, the aim of the study was to determine metabolic differences between both types of ALS, as well as the possible relationship with muscle function. Materials and Methods: A descriptive, analytical, quantitative, and transversal study was carried out in hospitals and Primary Care centers in the region of Valencia, Spain. Fasting glucose and alkaline phosphatase (AP) levels in venous blood, muscle percentage, fat percentage, muscle strength (MRC scale), and functional capacity (Barthel Index) were measured in 31 patients diagnosed with ALS (20 with spinal onset ALS and 11 with bulbar onset ALS). A healthy control of 29 people was included. Results: No significant differences were observed in blood AP and glucose levels between spinal onset and bulbar onset ALS patients. However, a significant positive correlation was observed between the mean values of both substances in patients with spinal onset ALS. Moreover, a lower percentage of muscle mass and a higher percentage of fat mass were also seen in spinal ALS patients, who also presented lower muscle strength and lower functional capacity. Conclusion: The results of this study seem to point to a possible difference in the peripheral use of glucose between patients with bulbar onset ALS and spinal onset ALS, who appear to have possible insulin resistance. These metabolic differences could explain the lower muscle percentage and lower muscular function in spinal onset ALS patients, although further studies are required.

## 1. Introduction

Amyotrophic lateral sclerosis (ALS) is the most common neurodegenerative disease among those affecting motor neurons. Despite being considered a rare disease, the prevalence of ALS has increased due to an incidence increase in the last few years [1]. It is characterized by the selective death of motor neurons, and it can have a bulbar onset when it begins affecting the motor neurons in the medulla, or a spinal onset (limb onset) when it begins with a loss of strength and weakness in the extremities. Both types eventually lead to progressive paralysis of the voluntary muscles with progressive muscle waste, resulting in patient death [2] within 3 to 5 years [3]. However, the severity of the disease varies depending on the type of ALS, being more severe and with a more rapid progression when it has a bulbar onset [4]. These differences could be due to the pathogenesis of the disease.

At present, we know about oxidative stress, free radicals excess, abnormal accumulation of neurofilaments, and the excitotoxicity linked to an increase in the neurotransmitter glutamate, which will eventually lead to an alteration in the mitochondrial activity in the spinal cord. This alteration causes disequilibrium in the energy balance linked to lower activity of the enzymes of the mitochondrial electron transport chain in the spinal cord [5]. This mitochondrial energy disorder can also be linked to problems in glucose metabolism. At the same time, it has been seen that there is a risk of developing diabetes mellitus in patients with ALS, possibly because defects in the SOD1 gene are related to alterations in glucose metabolism, observed in bacteria and yeasts [6]. In turn, patients with diabetes have a higher risk of developing ALS [7]. However, the association between diabetes and ALS is controversial since some studies show that diabetes could be a protective factor against the disease [8]. Despite this, alterations in the glucose metabolism in ALS are evident and they could be one of the causes of the origin and progression of this fatal pathology [9].

On the other hand, this energy alteration could be different between bulbar and spinal onset ALS due to the differences seen in the metabolic and neuropsychological condition between both types of ALS [10,11]. As a consequence, metabolic differences related to the pathogenesis of the disease could be established, specifically, those associated with in glucose metabolism [12,13,14]. Along these lines, to ascertain glucose metabolism in the muscle, it is not only interesting to determine glucose blood levels but also glucose access to the cell. Thus, determining the values of Alkaline Phosphatase (AP) could be highly effective, since high levels may be associated with high bone destruction, on one hand [15], or biliary obstruction, on the other hand, if the AP and the gamma-glutamyl transferase (GGT) levels are also elevated [16]. However, if GGT is not elevated, or if there is evidence that the patient does not suffer from bone resorption, increased values of AP have been related to alterations in glucose metabolism, more precisely with insulin resistance, leading to alterations of the glucose metabolism by limiting glucose entry to the cell [14,17,18].

Muscle atrophy, characteristic of the disease, could be explained due to problems in peripheral glucose homeostasis associated with alterations in energy metabolism [19]. Regarding anthropometry, the disease is characterized by progressive deterioration with loss of lean mass [20], which, has a direct impact on the worsening of lung function [21]. Fat mass is also an important indicator in the prognosis of ALS, and greater survival has been seen in obese people with the disease [22]. Several studies also highlighted a prognostic role for body mass in ALS. In fact, a lower body mass index (BMI) in the presymptomatic phase or at the time of diagnosis predicts a shorter survival [23,24]. Along the same lines, ALS patients lose weight and body fat as the disease progresses, thus that fat deposits determine the progression of the disease [25]. All these anthropometric differences between both types of ALS could have an impact on muscle strength and, as a consequence, on functional capacity. In turn, these variables can have a direct relationship with the differences in severity and prognosis between both types of ALS, as previously mentioned.

Thus, based on the hypothesis that there are differences in the glucose metabolism in the CNS between bulbar and spinal onset ALS, the aim of the study is to establish whether there are differences in the peripheral use of glucose between bulbar onset and spinal onset ALS, and whether said differences could also result in muscle function differences in both types. 

## 2. Materials and Methods

### 2.1. Study Design

Descriptive, analytical, quantitative, and cross-sectional study.

### 2.2. Study Population

The sample was obtained from different Primary Care centers of the Region of Valencia (Spain). The selection criteria were the following:

Inclusion criteria: ALS patients (bulbar or spinal onset), over 18 years of age and with clear symptoms in the last 6 months. Exclusion criteria: Patients with diabetes mellitus type I or drug-induced, patients with glycosylated hemoglobin values between 5.7 and 6.4%, pregnant women or women who were breastfeeding, patients with bone pathologies, patients with swallowing problems, patients with hepatic disease with elevated hepatic markers 3 times above normal, patients with choledocholithiasis or gallbladder abnormalities, and patients treated with Riluzole.

The required sample size was calculated to detect the differences in the peripheral use of glucose between both types of ALS (bulbar and spinal). As information on this topic is scarce in the literature, we did not find a previous estimate of this difference. Therefore, we used our preliminary results to establish a hypothesis. Thus, in order to detect a difference of 7 points, with hypothetical standard deviations within groups of 8, a confidence level of 95%, and a power of 80%, 44 patients were necessary (22 in each group). However, given the low prevalence of the disease, the total number of patients with ALS in the region of Valencia was expected to be low, which would imply a correction by finite population in the estimation of the necessary sample size. According to a report by the Spanish Foundation for the Brain [26], the prevalence of ALS was estimated between 2 and 5 out of every 100,000 population. In neighboring regions like Catalonia, this prevalence has been estimated at 5.4 out of every 100,000. It seems reasonable to assume a prevalence of around 5 out of every 100,000 in our region. Based on this and considering that the region of Valencia has a population of 2,548,000 people, it was estimated that there would be approximately 127 people with ALS. As it is a small (finite) population, we made the correction of the sample size as follows:

(1) $$n_{e}=\frac{n}{1+\frac{n}{N}}=\frac{44}{1+\frac{44}{127}}=32.678$$

Therefore, the sample size was finally estimated as 32 patients. Moreover, a healthy control of 29 people was included. They were informed about the aims of the study, they signed a consent form, and their participation was voluntary.

### 2.3. Methods, Devices, and Procedures

**Figure 1:**
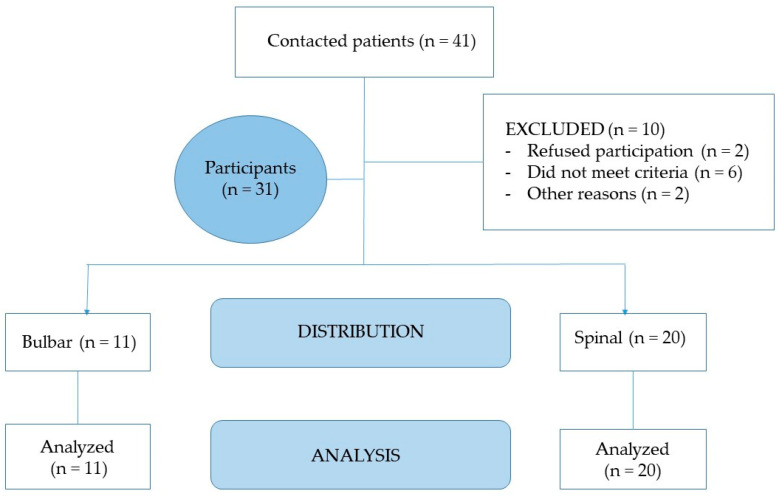
Consort flow.

The recruitment of patients (Figure 1) was carried out in September and October 2020, and all patients signed an informed consent after receiving written information about the study. We registered their sociodemographic data (age, sex) and health data (duration of symptoms and type of ALS).

Based on the selection criteria, a fasting blood sample was obtained from each patient (at 9 a.m.). Immediately after blood extraction, the glucose level of each patient was measured using a GM9 automatic analyzer from Analox and also their AP level using the Alkaline Phosphatase Liquiform kit from Labtest (Ref. 79-4/30).

In order to determine the muscle mass percentage of the patients, the variables considered were weight, height, brachial circumference (BC), triceps skinfold (TS), arm muscle circumference (AMC), and calf circumference (CC). The AMC was carried out following the International Society’s criteria for the Advancement of Kinanthropometry (ISAK) [27]. In addition, TS measurements and CC [28] were also obtained. These measurements were taken using calibrated instruments like the SECA 700 mechanical scale with attached SECA height rod, a scale with a ±100 g precision and maximum capacity of up to 220 kg, and a height rod with a ±0.1 cm precision [27]. Regarding patients who could not stand or who were bedridden, indirect calculation formulas were used to obtain an estimated weight and height [29]. A metal, narrow, and inextensible anthropometric tape, Lufkin W606PM, with an accuracy of ±0.2 mm, was used to measure CC and AMC [27]. To obtain the arm muscle circumference, the following formula was used [30].

(2) AMC=BC(cm)−(3.14×TS(cm))

In order to measure the triceps skinfold, a Langer skinfold caliper was used, with a precision of ±1 mm [30].

Body Mass Index (BMI) was calculated with the weight (kg) divided by the square of the height in meters (m^2^).

The ALS Functional Rating Scale (ALSFRS-R) was used to measure 12 aspects of physical function in ALS patients in 4 domains, designed to assess disabilities according to the activities of daily living (ADL) [31].

Barthel Index was also used to give different scores according to the patient’s abilities to carry out basic activities of daily living (BADLs). It includes 10 personal activities: Feeding, personal toileting, bathing, dressing and undressing, getting on and off a toilet, controlling bladder, controlling bowel, moving from wheelchair to bed and returning, walking on a level surface (or propelling a wheelchair if unable to walk), and ascending and descending stairs, each of them receiving a score of zero, if the person was unable to perform the task, or a variable score between 5, 10, and 15 points, which reflected independence or intervals of relative autonomy for the tasks. The score given to each activity was based on the time and amount of physical assistance needed if the patient was unable to perform the activity. Full credit was not given for an activity if the person needed minimal help and/or supervision [32].

Medical Research Council Scale (MRC) directly determines muscle strength. This scale was used for 8 different muscles: Biceps, triceps, quadriceps, and tibialis. The MRC rating system provided the following scores: 0, no contraction; 1, flicker or trace of contraction; 2, active movement, with gravity eliminated; 3, active movement against gravity; 4, reduced muscle strength, but movement against resistance is possible; 5, normal strength. To calculate the index of the total MRC scale, each muscle was assigned a number that progressively increased from 0 (0 in the MRC scale) to 10 (5 in the MRC scale). The total MRC index per patient corresponded to the sum of the numbers given to the 8 muscles [33].

King’s ALS clinical staging uses five stages, from 1 to 5, and was based on disease burden as measured by clinical involvement and significant feeding or respiratory failure, with stage 1 being symptom onset and stage 5 being death. The King’s staging system was not based on ALSFRS-R scores but can be estimated from them with 92% concordance [34].

### 2.4. Ethical Aspects

This study was carried out according to the ethical criteria established in the declaration of Helsinki [35].

### 2.5. Statistical Analysis

SPSS version 24.0 and Microsoft Excel 2016 were used to analyze the data obtained. A descriptive analysis was carried out, showing the absolute (*n*) and relative (%) frequencies for categorical variables and the mean and standard deviation for quantitative data. The Kolmogorov–Smirnov test was used to test the normality of the distribution. The chi-square test was used to analyze the relationship between categorical variables. To analyze the possible differences in age, time since symptom onset, glucose level, and AP level between the bulbar and the spinal ALS groups, the non-parametric Mann–Whitney U test was applied, given the lack of normality in the distribution. The Spearman’ correlation coefficient was used to analyze the correlations between age, glucose level, and AP level. The significance level was set at *p* < 0.05 in all cases.

## 3. Results

After the selection criteria, and because it is a disease with very low prevalence [36], the final population sample of ALS patients for the study was 31 patients, where 64.5% were men. In the total sample, patient ages were between 45 and 82 years, with a mean of 59.48 ± 9.97. The time since the onset of symptoms was 40.96 ± 18.22 months. 20 patients had spinal onset ALS and 11 had bulbar onset ALS. There was no difference in sex, age, weight, BMI, time since symptom onset, or functional capacity between them as determined by the ALS Functional Assessment Scale (ALSFRS). Furthermore, when comparing disease progression using the King’s system in stage 1 or 2 ALS patients, there were no significant differences between both groups.

In the healthy control, when comparing the same variables with bulbar and spinal ALS, there were no significant differences, except for weight since healthy patients presented higher values than bulbar and spinal ALS patients. All results are described in Table 1.

Regarding the results obtained for glucose and AP, their average values were within the reference range, with values of 96.62 ± 10.03 for glucose and 78.44 ± 30.05 for AP. When comparing the values of both variables according to functional capacity between patients above or below 40 on this scale, no significant differences were observed for either of the variables (Table 2).

No significant differences were observed either when comparing the values of glucose and AP between bulbar and spinal onset ALS patients. AP and glucose values in both types of ALS were also compared with the healthy control, and no significant differences were observed (Table 3).

**Figure 2:**
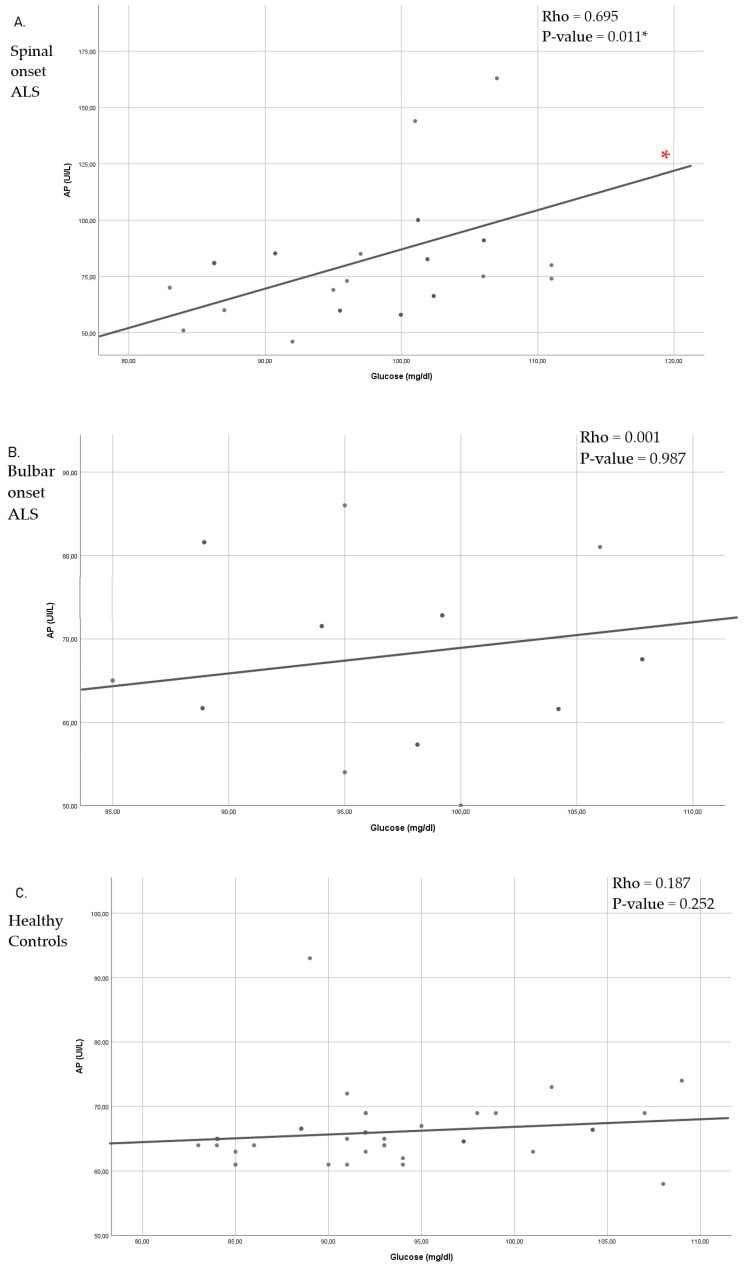
Spearman’s correlation coefficient between Glucose and AP, in Spinal onset ALS (**A**), Bulbar onset ALS (**B**) and Healthy control (**C**). ALS: Amyotrophic lateral sclerosis; AP: Alkaline phosphatase; * *p* < 0.05.

On the other hand, when analyzing the possible correlations between glucose and AP, a significant positive correlation was observed between them in spinal onset ALS patients. However, this correlation was not observed in bulbar ALS or the health control. (Figure 2).

As a consequence of the results in Figure 2, which could indicate certain insulin resistance in spinal ALS, muscle activity was assessed by comparing it between both groups of patients. When analyzing the muscle percentages of both types of ALS, a significantly higher percentage of muscle mass could be observed in patients with bulbar onset ALS compared to patients with spinal onset ALS. In addition, patients with spinal onset ALS had a significantly higher percentage of fat mass, as well as lower muscle strength (MRC) and functional capacity (Barthel index) (Table 4).

## 4. Discussion

To date, the energy of metabolic alterations at the energy level in ALS remains unclear. However, many authors suggest that they could occur before the loss of motor neurons and long before the appearance of motor symptoms. These alterations have been seen at the cerebral level [37] and the muscle [38,39,40], and their origin could be one of the aspects that explains the bulbar or the spinal onset of the disease.

These metabolic alterations include problems in glucose metabolism observed in the hSOD1^G93A^ mouse model pf the disease and evidenced by a decrease in the up-take of (3-^13^C) pyruvate in the Krebs cycle [9], which contributes to hyperexcitability [41]. In turn, in the study by Pradat et al., 2010, abnormal glucose tolerance was seen in non-diabetic ALS patients when compared to healthy patients, which was accompanied by higher levels of free fatty acids in the blood that indicate insulin resistance [14]. These results seem to be in line with the conclusions recently published by Cheng et al., which show fasting glucose levels are higher in patients with ALS compared to healthy people [42]. However, neither of the two studies made a distinction between patients with bulbar or spinal onset ALS. In our study, non-diabetic patients were compared with both types of ALS, showing that there were no significant differences, as it occurs when comparing glucose and AP levels with the healthy control. Nevertheless, positive significance is observed in the correlation between both molecules only in spinal onset ALS, which could indicate that there is some insulin resistance for this type of disease. Insulin resistance delays glucose clearance, observed after performing the glucose tolerance test in transgenic mouse model MLC/SOD1^G93A^, which was associated with a lower number of Glut-4 receptors in the muscle fibers [43]. The Glut-4 receptors are responsible for muscle glucose uptake and are related to insulin sensitivity since lower expression of this receptor increases hormone resistance [44]. As a consequence, in the animal model SOD1-G86R, early inhibition of the glycolytic capacity of muscle fibers has been observed, with a progressive inhibition of phosphofructokinase 1 and the induction of pyruvate dehydrogenase kinase 4 expression (strongly inactivating pyruvate dehydrogenase that way) [38]. Therefore, the restriction of glucose uptake would lead to hypometabolism in the cells of the skeletal muscle, which would explain the significant decrease in skeletal muscle [45] that we found in spinal onset ALS patients. Although these results must be confirmed with further studies, as a consequence of this apparent insulin resistance, we can observe significant differences muscle differences between bulbar and spinal onset ALS patients. Spinal patients present a higher percentage of muscle mass coinciding with the results already published by Salvioni C.C. et al., who observed greater muscle circumference in the arm in bulbar ALS. Furthermore, in this same study, it was also seen that the levels of fat mass were higher in these patients than in those with bulbar onset ALS [46], which coincides with our results. This fact could also be a consequence of muscle atrophy, since skeletal muscle is essential for the metabolism of dietary fat, with the fatty acids derived from fat being the main source of energy for resting and working muscle [47,48]. In this sense, PPAR are critical regulators of metabolic genes in the skeletal muscle [49]. Among these, the PPARα participates in the transcription of genes that are necessary for the absorption or oxidation of fatty acids, thus that the activation of PPARα, induces the utilization of fatty acids in skeletal muscle [50]. Therefore, the reduction in muscle mass throughout the ALS disease can in turn decrease the ability to metabolize fatty acids, increasing the percentages of fat mass. However, it should be noted that fat has a protective effect in ALS [22], an aspect that could be linked to lower severity of spinal onset ALS that presents a better prognosis [4]. 

There is a direct relationship between muscle mass and muscle strength. Loss of muscle strength is mainly explained by a reduction in the intrinsic capacity of skeletal muscle fibers to generate force [51,52], which, in relation to ALS, type I fibers would presumably be the main responsible for the loss of strength since they are more susceptible to denervation-induced atrophy [53]. Thus, it can be observed how, for example, that older skeletal muscle shows a reduction in its intrinsic force-generating capacity of approximately 34% [54]. This relationship between loss of muscle mass and muscle strength is seen in our study population because spinal patients with a lower percentage of muscle mass also have less muscle strength.

On the other hand, alterations in the structure and function of the neuromuscular junction, as well as fat infiltration, affect muscle strength and energy production [55]. Therefore, there is a direct relationship between muscle strength and the presence of fat mass, with functionality loss [56,57], which could explain why in spinal onset ALS patients, we have observed a lower functional capacity in the Barthel Index.

Nonetheless, regarding the limitations of the study, our initial hypothesis could be an epiphenomenon of a pathogenic process that is not peripheral in nature, so more robust research will be necessary. It is also important to point out that the differences observed in the levels of AP and glucose are not significant between both types of ALS. This could be due to the low population sample (despite an increase in the incidence, the amount of people who suffer from ALS is relatively small) since based on the typical deviations observed in our sample, with a 95% confidence interval and 80% power, for a population of 90 patients in each group, the differences would have been significant. It should be considered that this is a pilot study, and further research is required to confirm these differences and in order to understand the complexity of this neurodegenerative disease more in-depth. Therefore, it will be necessary to increase the sample size in order to obtain more relevant results. Finally, as an important limitation, there is a lack of data about the involvement of the central motor system, which is a requirement for diagnosis and also has an impact on peripheral motor/muscular disorder.

## 5. Conclusions

In conclusion, the results of this study seem to point to a possible difference in the peripheral use of glucose between patients with bulbar and spinal onset ALS. In patients with spinal onset ALS, we find a possible insulin resistance determined by the measurement of AP and glucose in venous blood, and this could explain the lower muscle percentage and lower muscle function (with less strength and functional capacity). However, further studies are needed to confirm these conclusions.

## Figures and Tables

**Table 1:** Baseline characteristics of the sample.

		Bulbar Onset ALS *N* = 11	Spinal Onset ALS *N* = 20		Bulbar Onset ALS *N* = 11	Healthy Control *N* = 29		Spinal Onset ALS *N* = 20	Healthy Control *N* = 29	
		Frequency (%)	Frequency (%)	Chi-Square (*p*-Value ^a^)	Frequency (%)	Frequency (%)	Chi-Square (*p*-Value ^a^)	Frequency (%)	Frequency (%)	Chi-Square (*p*-Value ^a^)
Sex	Female	4 (36.36%)	7 (35%)	0.040 (0.84)	4 (36.36%)	10 (34.48%)	0.012(0.91)	7 (35%)	10 (34.48%)	0.001 (0.97)
	Male	7 (63.64)	13 (65%)		7 (63.64)	19 (65.51%)		13 (65%)	19 (65.51%)	
King’s ALS clinical staging	1	6 (54.5%)	11 (45.5%)	0.021 (0.886)						
	2	4 (36.3%)	11 (63.7%)							
		Mean (SD)	Mean (SD)	Z (*p*-Value ^b^)	Mean (SD)	Mean (SD)	Z (*p*-value ^b^)	Mean (SD)	Mean (SD)	Z (*p*-Value ^b^)
Age(years)		60.80 (11.62)	58.99 (9.46)	−0.269 (0.79)	60.80 (11.62)	59.8 (8.71)	0.789 (0.423)	58.99 (9.46)	59.8 (8.71)	1.856 (0,06)
Weight(kg)		68.0 (10.5)	69.5 (8.6)	−0.421 (0.674)	68.0 (10.5)	82.93 (13.88)	2.584 (0.009 **)	69.5 (8.6)	82.93 (13.88)	3.023 (0.002 **)
BMI		23.9 (2.5)	24.7 (2.8)	−0.774 (0.439)	23.9 (2.5)	28.62 (3.76)	1.53 (0.126)	24.7 (2.8)	28.62 (3.76)	0.834 (0.41)
Time since symptom onset (months)		43.87 (22.95)	39.63 (15.98)	−0.398 (0.699)						
ALSFRS-R		36.7 (6.5)	33.9 (9.9)	−0.405 (0.687)						

ALS: Amyotrophic lateral sclerosis; SD: Standard deviation. BMI: Body Mass Index. ALSFRS: Amyotrophic Lateral Sclerosis Functional Rating Scale. ^a^ Chi-square. ^b^ Mann–Whitney U test. ** *p* < 0.05.

**Table 2:** Mean differences in glucose and AP between ALS patients according to ALSFRS-R score.

	ALS Patients ALSFRS-R Score <40 *N* = 18		ALS Patients ALSFRS-R Score >40 *N* = 13		Mann-Whitney U TEST	
	Mean	SD	Mean	SD	Z	*p*-Value
Glucose (mg/dL)	98.8	10.1	93.6	8.48	1.279	0.200
AP (UI/L)	79.5	34.8	75.5	24.2	−0.310	0.756

Normal values: Glucose: 70–110 mg/dL, fasting; AP: 27–100UI/L; ALS: Amyotrophic lateral sclerosis; ALSFRS-R: ALS Functional Rating Scale AP: Alkaline phosphatase; SD: Standard deviation.

**Table 3:** Mean differences in glucose and AP between healthy people (healthy control), and patients with bulbar and spinal ALS.

	**Bulbar Onset ALS** ***N* = 11**		**Spinal Onset ALS** ***N* = 20**		**Mann-Whitney U Test**	
	**Mean**	**SD**	**Mean**	**SD**	**Z**	***p*-Value**
Glucose (mg/dL)	92.82	8.6	98.60	10.5	−1.122	0.287
AP (UI/L)	66.93	14.2	84.35	34.6	−0.994	0.329
	**Healthy Control** ***N* = 29**		**Bulbar Onset ALS** ***N* = 11**			
	**Mean**	**SD**	**Mean**	**SD**	**Z**	***p*-Value**
Glucose (mg/dL)	93.03	7.15	92.82	8.6	−0.093	0.926
AP (UI/L)	66.17	6.49	66.93	14.2	−0.088	0.930
	**Healthy Control** ***N* = 29**		**Spinal Onset ALS** ***N* = 20**			
	**Mean**	**SD**	**Mean**	**SD**	**Z**	***p*-Value**
Glucose (mg/dL)	93.03	7.15	98.60	10.5	−0.870	0.384
AP (UI/L)	66.17	9.49	84.35	34.6	−2.028	0.243

Normal values: Glucose: 70–110 mg/dL, fasting; AP: 27–100UI/L; ALS: Amyotrophic lateral sclerosis; AP: Alkaline phosphatase; SD: Standard deviation.

**Table 4:** Functional profile and anthropometric data according to Amyotrophic lateral sclerosis (ALS) subtype.

	Whole Sample ALS *N* = 31 Mean ± SD (95% CI)	Bulbar Onset ALS *N* = 11 Mean ± SD (95% CI)	Spinal Onset ALS *N* = 20 Mean ± SD (95% CI)	Mann-Whitney U Test	
				Z	*p*
Barthel index	75.3 ± 22.9 (63.8–86.7)	94.0 ± 13.4 (77.3–110.6)	68.1 ± 22.0 (54.7–81.3)	−2.265	0.026 *
Fat %	18.2 ± 3.8 (16.4–19.9)	15.1 ± 3.2 (11.7–18.4)	19.4 ± 3.5 (17.4–21.3)	−2.180	0.029 *
Muscle %	36.8 ± 3.1 (32.2–40.6)	39.9 ± 2.2 (37.6–42.2)	35.6 ± 2.5 (34.2–37.1)	−2.725	0.006 **
MRC	58.2 ± 16.4 (50.7–65.7)	73.0 ± 7.1 (65.5–80.4)	52.3 ± 15.3 (43.8–60.8)	−2.618	0.009 **

MRC: Medical Research Council Scale; ALS: Amyotrophic lateral sclerosis; SD: Standard deviation; * *p* < 0.05; ** *p* < 0.01.
